# RFE-YOLO: A Lightweight Receptive Field-Enhanced Network for UAV Imagery Object Detection

**DOI:** 10.3390/s26092903

**Published:** 2026-05-06

**Authors:** Yimo Peng, Xiangyu Ge

**Affiliations:** College of Geography and Remote Sensing Science, Xinjiang University, Urumqi 830017, China; 20231210213@stu.xju.edu.cn

**Keywords:** small object detection, remote sensing image, deep learning, feature fusion

## Abstract

Object detection in unmanned aerial vehicle (UAV) remote sensing imagery remains a formidable challenge due to the diminutive scale of targets, complex background clutter, and extreme variability in target morphology. Standard convolutional neural networks typically suffer from irreversible fine-grained information loss during downsampling, as strided operations discard critical spatial details essential for the localization of tiny objects. To address these issues, we propose RFE-YOLO, a lightweight receptive field-enhanced network specifically tailored for high-precision small object detection in UAV scenarios. First, the Cross-Scale Receptive Field Enhancement (CSRE) module is designed to mitigate intrinsic information loss by integrating space-to-depth convolution (SPD-Conv), which preserves spatial details by migrating them into the channel dimension. This module further employs an energy-based adaptive weight generation mechanism to distinguish target signals from environmental noise. Second, this paper proposes the C3k2-Dynamic Inception Mixer Block (C3k2-DIMB), which adaptively captures anisotropic features—such as slender vehicles—via dynamic kernel weighting and multi-shape inception kernels. Third, the Shuffled Upsampling for Resolution Enhancement (SURE) module is introduced to maintain spatial fidelity during resolution recovery, utilizing a channel shuffle mechanism to overcome information isolation. Finally, the Multi-feature Fusion Module (MFM) replaces conventional static concatenation with a dynamic softmax-based competition mechanism, effectively bridging the semantic gap between multi-level features while suppressing background distractors. Experimental results on the VisDrone dataset demonstrate that RFE-YOLO significantly enhances the representation capability for small objects. Specifically, the proposed model achieves a state-of-the-art $mAP_{50}$ of 42.70%, representing a substantial 9.3% improvement over the baseline YOLO11n. Furthermore, our architecture maintains an exceptionally lightweight profile with only 1.91 M parameters, demonstrating that high-precision detection can be achieved through structural intelligence rather than excessive parameter scaling. This makes RFE-YOLO highly suitable for real-time inference on edge-deployed UAV platforms.

## 1. Introduction

In recent years, with the application and popularization of drones in both civilian and military fields, drone images have been widely used in intelligence reconnaissance, remote sensing mapping, traffic monitoring, and other areas [1,2,3,4]. The artificial intelligence image processing technology represented by deep learning has made significant progress, enabling rapid development of remote sensing target detection algorithms based on convolutional neural networks (convolutional neural network, CNN). These algorithms have overcome the accuracy and speed bottlenecks of traditional manual image feature extraction methods. Deep learning [5,6] uses an end-to-end feature learning mechanism to automatically extract multi-level and high-semantic abstract features, overcoming the limitations of traditional methods that rely on handcrafted features. It can more accurately capture the texture, shape, and context information of targets in complex scenes, and deep learning models [7,8,9] (such as CNN, Transformer) inherently have the ability to handle multi-scale and multi-directional targets. Through structures such as feature pyramids and attention mechanisms, it effectively solves the problems of small target detection and rotation target positioning. Its robustness against complex background interference (such as clouds, shadows, similar ground features) is stronger. Combined with data augmentation and adaptive fusion strategies (such as multimodal data fusion), it significantly improves detection accuracy and generalization ability. These advantages make deep learning the core technology for remote sensing target detection, promoting the transition from traditional rule-driven to data-driven target detection, and it is widely applied in various scenarios, such as maritime rescue, reconnaissance and warning, and urban planning. By leveraging the high-resolution, multi-functional, and real-time data collection capabilities of drones, researchers aim to overcome the challenges brought by traditional remote sensing methods and explore new possibilities in target detection and analysis. However, the images and videos captured by drones differ significantly from those captured from the human eye’s perspective. Drone images typically have the characteristics of an aerial view, with variable angles and heights. This leads to challenges such as uneven target distribution, small target proportions, complex backgrounds, and susceptibility to weather conditions. Moreover, due to the inherent characteristics of common target detection methods such as convolutional neural networks and self-attention mechanisms—specifically, the insufficient feature representation caused by traditional downsampling methods (stride convolution, pooling), where the targets occupying a limited pixel area exhibit the least texture and shape information; the progressive feature degradation caused by the hierarchical structure of the deep network; and the limitations in the performance of upsampling of deep semantic features and the fusion of shallow features—it cannot balance high semantic information and high spatial resolution. Specifically, traditional downsampling methods (stride convolution, pooling) periodically discard pixel data, leading to irreversible information attrition that erodes the structural integrity of micro-scale targets. This creates a fundamental conflict: expanding the effective receptive field often dilutes the fine-grained details necessary for precise localization. Moreover, the onboard computing platform has limited computing power, requiring careful trade-offs between detection accuracy and computational efficiency, which requires the model to have real-time processing capabilities when running on embedded aerial platforms. These interrelated challenges jointly define the unique problem space of the drone-based detection system, requiring specialized architectural considerations beyond traditional computer vision methods.

In summary, UAV-based detection faces three critical logical bottlenecks that current CNN architectures fail to address: (1) Irreversible Information Attrition, where standard downsampling (stride convolution/pooling) periodically discards pixel data, leading to insufficient feature representation for minute targets; (2) Anisotropic Geometric Distortion, where variable flight altitudes and angles create targets with high aspect ratios that static isotropic kernels cannot effectively model; and (3) Semantic–Spatial Conflict, where traditional upsampling and static fusion lead to semantic blurring and indiscriminate noise propagation from complex terrestrial backgrounds. These challenges require a specialized “Preservation–Adaptation–Fusion” architecture to ensure high-precision detection on edge-deployed platforms with limited computing power.

In the field of unmanned aerial vehicle (UAV) target detection, methods based on convolutional neural networks (CNNs) have become the mainstream research framework. Academically, they are typically classified into two-stage and one-stage detection paradigms [10]. The widely adopted two-stage detection methods, such as Faster R-CNN [11], Mask R-CNN [12], Cascade R-CNN [13], and Dynamic R-CNN [14], first generate candidate regions in the image, then achieve the classification and localization of the target regions through feature extraction, and finally output the category labels and spatial coordinates of the detected targets. In contrast, single-stage detectors like YOLO [15] and FCOS [16] model the detection task as a unified regression problem. These methods usually rely on anchor box mechanisms to simultaneously complete target localization and classification without relying on region proposals, thereby simplifying the detection process and improving overall efficiency and structural simplicity. However, compared to conventional images, UAV images often exhibit significant scale and position variations in targets, especially a large number of small-sized and densely distributed targets to be detected; these dense small targets are more prominent under different resolutions, further exacerbating the diversity of scale and spatial distribution in UAV images. Moreover, due to differences in flight height, shooting angle, and imaging distance, UAV images also have significant differences in perspective, resolution, and other aspects from conventional images.

These interrelated challenges, along with the limitations of existing methods, jointly define the unique issues of the unmanned aerial vehicle-based detection system, requiring specialized architectural considerations beyond traditional computer vision methods.

The primary objective of this study is to develop a lightweight yet powerful detector specifically optimized for small object detection in complex UAV environments. The main contributions of this paper are summarized as follows:

1. This paper proposes RFE-YOLO, a lightweight receptive field-enhanced network. The designation “RFE” reflects the core architectural philosophy of the network: dynamically modulating and expanding the effective receptive field to capture multi-scale structural cues and anisotropic features, which are frequently compromised in standard lightweight architectures. RFE-YOLO establishes a robust pipeline from lossless feature preservation to dynamic semantic integration, specifically addressing the unique “information isolation” and “semantic blurring” challenges of UAV-based remote sensing.
2. This paper designs the Cross-Scale Receptive Field Enhancement (CSRE) module. By integrating SPD-Conv with an energy-based adaptive attention mechanism ($e_{t}$), the module mitigates irreversible information loss during downsampling and purifies target signals from cluttered terrestrial backgrounds.
3. This paper introduces the Dynamic Inception Mixer Block (C3k2-DIMB), which utilizes multi-shape inception kernels and a dynamic kernel weighting (DKW) path. This allows the network to adaptively modulate its receptive field to capture the anisotropic features of targets with variable aspect ratios and orientations.
4. This paper develops the Shuffled Upsampling for Resolution Enhancement (SURE) module and the Multi-feature Fusion Module (MFM). These components collectively enhance spatial fidelity during resolution recovery through a channel shuffle mechanism and bridge the semantic gap between multi-level features via a dynamic softmax-based competition strategy.
5. Extensive experiments on the VisDrone [17] dataset demonstrate that the proposed RFE-YOLO achieves a superior trade-off between detection accuracy and computational efficiency, proving its effectiveness for real-time deployment on edge-constrained UAV platforms.

## 2. Related Works

This section provides an introduction to three related directions that are closely related to our approach. These works include target detection in drone images, as well as typical multi-scale feature fusion methods and small target detection methods used in the target detection task.

### 2.1. Object Detection in UAV Imagery

With the widespread proliferation of unmanned aerial vehicles (UAVs), UAV-based object detection has emerged as a significant focal point in computer vision research. Leveraging their precision, high efficiency, and extensive monitoring capabilities, UAVs have substantially advanced the development of remote sensing object detection. In the realm of conventional remote sensing, mainstream methodologies are predominantly driven by convolutional neural networks (CNNs), which are categorized into two-stage detectors [11,12,13] and one-stage detectors [18,19]. Both paradigms have demonstrated superior performance in generic natural scene benchmarks.

Nevertheless, compared to natural imagery, UAV-captured images exhibit unique characteristics that limit the direct applicability of traditional CNN-based detectors. Specifically, UAVs frequently operate at varying altitudes and perspective angles, resulting in datasets characterized by ubiquitous small objects and congested spatial distributions.

In recent years, numerous specialized detection methods tailored for UAV imagery have surfaced. For instance, Lu et al. [20] introduced a hybrid CNN–Transformer architecture incorporating cross-shaped window transformations, hybrid patch embeddings, and slicing-based inference mechanisms to mitigate the challenges of complex backgrounds and non-uniform object distributions. Wang et al. [21] enhanced classification features via selective rotating convolution kernels, thereby optimizing feature representation for objects with high aspect ratios and bolstering oriented object detection through the integration of classification and regression features. Additionally, Ding et al. [22] proposed an attention module termed OSAM, which utilizes an efficient parameter design to capture discriminative spatial features. Furthermore, Yan et al. [23] presented an enhanced YOLOv10 framework optimized for UAV platforms; by integrating adaptive convolutions, multi-scale feature aggregation, and refined bounding box loss functions, this method significantly improves the detection precision and robustness for small objects in cluttered environments.

Despite these advancements, the detection accuracy for multi-scale objects remains constrained, primarily due to the suboptimal fusion and utilization of multi-scale features. Representative UAV datasets, such as VisDrone2019 [17], focus on urban traffic scenarios comprising ten categories (e.g., pedestrians and vehicles) with stark disparities in scale and position. For instance, pedestrian instances often occupy less than 6% of the image area, whereas vehicles may exceed 20%. Such extreme scale diversity necessitates more potent multi-scale feature processing capabilities to ensure accurate identification across varying object dimensions.

### 2.2. Multi-Scale Feature Integration Approaches in UAV-Based Object Detection

In convolutional neural network (CNN)-based UAV object detection, the feature pyramid (FP) has emerged as a pervasive strategy for integrating multi-scale features. Among various implementations, the Single-Shot MultiBox Detector (SSD) [19] and the Feature Pyramid Network (FPN) [24] represent two paradigmatic architectures. SSD constructs a feature hierarchy by leveraging feature maps from diverse CNN layers to simultaneously execute object classification and bounding box regression. This methodology heavily relies on deep convolutional features, which excel at capturing rich contextual information.

Nevertheless, UAV imagery is characterized by a high density of small objects with minimal pixel occupancy and sparse visual cues. High-level convolutional layers tend to extract abstract global semantics, which are inherently limited in preserving the subtle textures and localized details required to resolve such congested small instances. Consequently, SSD often fails to achieve optimal performance when confronted with the specific demands of small object detection in aerial perspectives.

To circumvent these limitations, the Feature Pyramid Network (FPN) significantly bolsters detection performance by efficiently incorporating high-resolution shallow features. FPN adopts a top-down architecture that facilitates the interaction between detail-rich shallow features and semantic-heavy deep features. Specifically, deep-layer features are upsampled and merged with shallow counterparts via lateral connections, thereby constructing multi-scale representations that possess both potent semantic discriminability and precise spatial localization. This architectural design empowers models to effectively navigate the typical challenges of UAV imagery, such as extreme scale variation and diverse spatial distributions. As a result, FPN has become a cornerstone for enhancing the precision and robustness of UAV detection tasks. Furthermore, HR-FPN [25] advances this paradigm by progressively aligning and fusing high-resolution features, effectively improving small object detection accuracy while maintaining a lightweight computational overhead.

### 2.3. Small Object Detection

According to the MS COCO dataset criteria, objects with an area less than or equal to 32×32 pixels are categorized as small objects. The AI-TOD [26] benchmark provides a more granular classification: objects with widths ranging from 2 to 8 pixels are defined as “very tiny”, those between 8 and 16 pixels as “tiny”, and those from 16 to 32 pixels as “small”.

To address the challenges posed by these scales, various methodologies have been proposed. Zhu et al. [27] leveraged Multi-head Self-Attention (MHSA) to capture global dependencies, thereby enhancing the model’s capacity to handle objects across diverse scales, including those of diminished size. Furthermore, super-resolution (SR) techniques have been integrated to narrow the feature representation disparity between small and large-scale objects. By learning high-resolution feature mappings, these methods bolster detection accuracy for small instances. For instance, Bai et al. [28] introduced a Multi-task Generative Adversarial Network (MTGAN) specifically designed to super-resolve Region of Interest (ROI) patches. Similarly, Zhang et al. [29] proposed an auxiliary super-resolution branch to enable the network to learn fine-grained structural details of small objects. Moreover, recent studies in industrial defect detection have highlighted that integrating receptive field expansion with multi-scale feature interaction is essential for resolving minute structural details. Zhu et al. [30] developed a subtle defect detection network (PDE-YOLO) to accurately identify minute anomalies on PCB surfaces by optimizing feature extraction for fine-grained details. Additionally, Li et al. [31] addressed the challenges of long-tail and multi-scale scenarios in surface defect detection, further demonstrating the necessity of robust feature representation and adaptive interaction when handling targets with extreme scale variations. Apart from architectural optimizations, data-level strategies such as tiling or Slicing-Aided Hyper Inference (SAHI) [32] have been widely adopted to resolve the micro-target detection bottleneck in large-scale UAV imagery. These methods involve partitioning high-resolution images into multiple overlapping patches to ensure that small targets possess a larger relative pixel area during inference. While effective in increasing precision, tiling-based approaches significantly amplify the computational latency due to the redundant processing of multiple tiles, which often conflicts with the real-time requirements of edge-deployed UAV platforms.

In the domain of remote sensing, the primary bottleneck for small object detection lies in the paucity of inherent feature information and the susceptibility to complex background interference. Consequently, contemporary research has pivoted towards strengthening the discriminative power of weak feature representations. Drawing inspiration from biological visual mechanisms, some studies have designed adversarial receptive field modules to heighten the contrast between targets and backgrounds [33]. Other works have focused on constructing sophisticated feature enhancement, fusion, and spatial context-aware modules to enrich feature semantics while suppressing environmental noise [29]. Additionally, some frameworks incorporate object reconstruction tasks during training and employ multi-receptive field adaptive modules to dynamically reinforce feature expression [34]. Recent advancements also include structural refinements to the feature pyramid and detection heads, such as the Denoising Feature Pyramid Network (DN-FPN) combined with Transformer-based detection heads [35].

### 2.4. Summary

In summary, existing research has significantly improved object detection performance in remote sensing images—particularly for small targets and complex backgrounds—through diverse strategies such as multi-level feature fusion, attention mechanisms, and specialized module designs. However, these approaches still face several inherent challenges. For instance, multi-scale feature fusion architectures (e.g., FPN, PANet [36]) heavily rely on standard downsampling operations (e.g., stride convolution or pooling). These operations reduce feature map resolution while often introducing feature misalignment, which is particularly detrimental to the precise localization of minute objects. Furthermore, while dynamic attention mechanisms (e.g., CBAM [37], Coordinate Attention [38]) effectively enhance feature representation capabilities, their ability to model spatial structural context remains limited when handling targets with extreme aspect ratios or arbitrary orientations (e.g., bridges, ships). At the label assignment and loss function level, the inherent high sensitivity of traditional Intersection over Union (IoU) and its variants on minute objects also limits further improvements in detection performance.

## 3. Materials and Methods

The definitions in Section 3.2 establish the geometric scale of “small” and “tiny” objects; this research specifically focuses on semantic categories critical to UAV-based urban surveillance and remote sensing. Specifically, the study targets dynamic micro-scale entities such as pedestrians, bicycles, and motorcycles (which frequently appear as “tiny” objects in VisDrone), as well as structurally diverse objects including vehicles, ships, airplanes, and bridges (which exhibit extreme scale variations in UAVDT [39] and NWPU VHR-10 [40]). By concentrating on these categories, the proposed RFE-YOLO aims to resolve the detection challenges associated with objects that possess both limited pixel support and complex morphological features.

### 3.1. Network Architecture Overview

**Figure 1:**
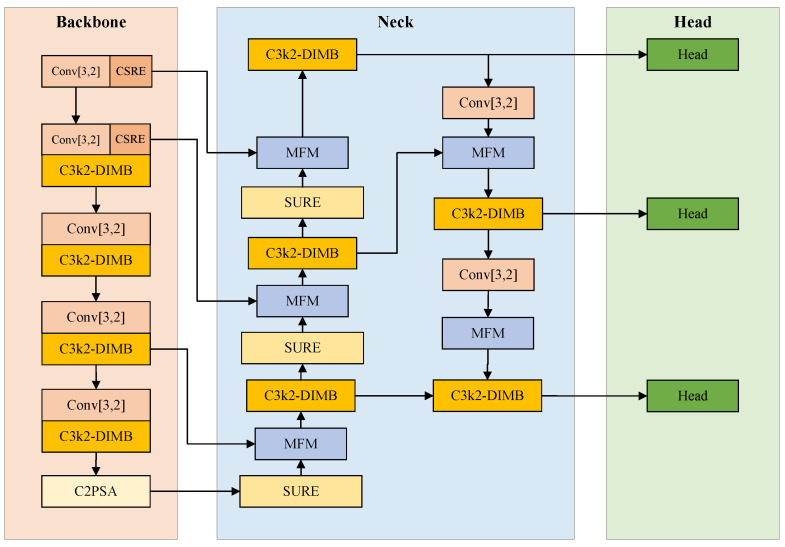
The overall architectural layout of RFE-YOLO, featuring the Backbone for lossless detail retention and the Neck for dynamic feature fusion.

The overall architecture of the proposed RFE-YOLO is illustrated in Figure 1. To resolve the identified sensing bottlenecks, RFE-YOLO establishes a hierarchical “Preservation–Adaptation and Fusion” pipeline. This research framework is designed to bridge the gap between pixel-level detail persistence and high-level semantic enhancement through two functional stages: a Feature-Preserving Backbone for lossless information migration and a High-Fidelity Neck for dynamic cross-scale integration.

Feature-Preserving Backbone: The backbone network is optimized for lossless detail extraction and morphological adaptation. Specifically, the CSRE module replaces standard downsampling to ensure fine-grained spatial information is migrated to the channel dimension via SPD-Conv [41] rather than being discarded. Simultaneously, the C3k2-DIMB acts as the core processor to adaptively extract features from targets with variable geometries.

High-Fidelity Neck: The neck network implements a high-fidelity integration strategy. The SURE module recovers spatial resolution with superior fidelity while breaking the information silos inherent in lightweight designs through channel shuffling. Subsequently, the MFM replaces static concatenation with a dynamic softmax-based competition mechanism to weight multi-level cues and suppress environmental noise.

Through this design, RFE-YOLO maintains a lightweight computational profile while significantly bolstering the discriminative power for targets with limited pixel support and complex morphologies.

### 3.2. Cross-Scale Receptive Field Enhancement Module

In the context of small object detection in UAV remote sensing imagery, traditional downsampling mechanisms—primarily relying on strided convolutions and pooling layers—pose significant challenges. Since minute targets typically occupy an extremely limited number of pixels, strided operations, which reduce spatial dimensions by periodically discarding pixel data, lead to the irreversible loss of fine-grained features and the degradation of structural integrity. Moreover, the static isotropic kernels employed in conventional modules lack the necessary flexibility to capture anisotropic features of targets with highly variable aspect ratios (e.g., vehicles). Concurrently, weak target signals are prone to being diluted by complex environmental background noise during the resolution reduction process. Furthermore, the inherent trade-off between expanding the effective receptive field and maintaining high spatial resolution prevents the network from preserving critical high-frequency spatial details required for precise localization while aggregating global semantic context.

**Figure 2:**
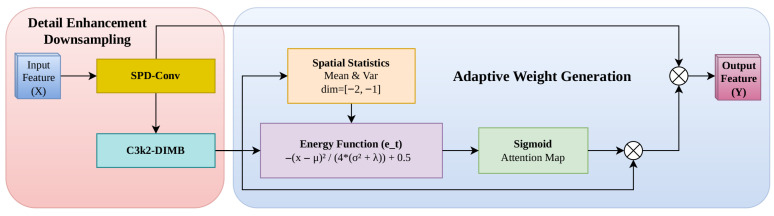
Detailed architecture of the proposed Cross-Scale Receptive Field Enhancement (CSRE) module. The module integrates two synergistic components: (1) Detail Enhancement Downsampling, which utilizes SPD-Conv to migrate spatial information into the channel dimension and C3k2-DIMB for dynamic feature extraction; and (2) Adaptive Weight Generation, which recalibrates feature responses using an energy-based attention mechanism ($e_{t}$) to purify target signals from complex environmental noise.

To mitigate the information attrition inherent in traditional downsampling, we propose the Cross-Scale Receptive Field Enhancement (CSRE) module. This section details the mathematical foundation and the refinement mechanism of the CSRE module; the structure of this module is shown in Figure 2.

To address the irreversible loss of fine-grained information, the module employs the space-to-depth (SPD) operation [41]. Given an input feature map $X\in R^{S\times S\times C_{1}}$, the SPD layer partitions X into $s^{2}$ sub-feature maps by periodically sampling pixels with a step size *s*. Following the space-to-depth transformation method proposed by Sunkara et al. [41], this transformation is defined as follows:

(1) $$X^{\prime }=f_{SPD}\left(X\right)\in R^{\frac{S}{s}\times \frac{S}{s}\times s^{2}C_{1}}$$

Unlike strided convolutions that discard periodic data, SPD-Conv migrates spatial pixels into the channel dimension, ensuring the structural integrity of minute targets is preserved.

To mitigate signal dilution, the module incorporates an adaptive weight generation path based on neuron energy. For a feature map $x\in R^{C\times H\times W}$, the module computes spatial statistics:

(2) $$\mu =\frac{1}{HW}\sum_{i=1}^{H}\sum_{j=1}^{W}x_{i,j},\;\sigma^{2}=\frac{1}{HW}\sum_{i=1}^{H}\sum_{j=1}^{W}{\left(x_{i,j}-\mu \right)}^{2}$$

Following the principle of linear separability, this module defines the energy function $e_{t}$ to measure the saliency of each neuron. This formulation is specifically derived from the SimAM parameter-free attention mechanism [42], which evaluates the importance of each neuron by solving an objective function based on the minimum energy:

(3) $$e_{t}\left(x_{i,j}\right)=-\frac{{\left(x_{i,j}-\mu \right)}^{2}}{4(\sigma^{2}+\lambda )}+0.5$$

where λ is a hyperparameter for numerical stability.

The energy function $e_{t}$ identifies target-relevant regions. Neurons deviating from the spatial mean signify high information content. This module maps these to attention weights $\alpha_{i,j}$ via a Sigmoid activation:

(4) $$\alpha_{i,j}=\sigma \left(\frac{1}{e_{t}\left(x_{i,j}\right)}\right)$$

This mechanism “purifies” the feature map by amplifying target signals while suppressing background noise, resolving the inherent conflict between receptive field expansion and high-resolution preservation.

The final attention weight $\alpha_{i,j}$ is derived by mapping the inverse of the energy function through a Sigmoid activation σ(·), ensuring the weights are constrained within (0,1). Let $x_{dimb}$ denote the output features from the C3k2-DIMB block. The output feature map Y is obtained by performing element-wise multiplication between the recalibrated features and the attention map, followed by a residual summation with the output of the *SPD-Conv* block to preserve the gradient flow:

(5) $$Y=\left(x_{dimb}⊗\alpha \right)⊕f_{res}\left(X_{spd}\right)$$

where ⊗ denotes element-wise multiplication, ⊕ represents the residual addition, and $f_{res}$ denotes a 1×1 convolutional projection (if necessary) to ensure channel dimensionality matching between the residual path and the attention-weighted path.

The physical rationale behind the CSRE module lies in its ability to mitigate the sampling-induced information decay inherent in standard downsampling.

From a signal processing perspective, traditional strided convolutions act as a non-ideal low-pass filter that periodically discards pixel data. According to the Nyquist–Shannon sampling theorem, when the sampling frequency (stride) is insufficient to capture the high-frequency components of minute targets, it leads to irreversible aliasing and information loss.

In contrast, the CSRE module implements a space-to-depth (SPD) transformation, which can be physically interpreted as a lossless spatial-to-channel migration. Instead of discarding pixels, CSRE reconfigures the spatial grid into the channel dimension, preserving the full energy of the original signal. Subsequently, an energy-based weighting mechanism is applied to recalibrate these channels. Physically, this ensures that the “high-frequency signatures” (e.g., edges and textures of small objects) are retained in the feature map, resolving the fundamental conflict between spatial resolution reduction and fine-grained detail preservation.

### 3.3. Dynamic Inception Mixer Block

Despite the success of conventional convolutional neural networks (CNNs), their reliance on static, fixed-size kernels imposes significant constraints when processing complex UAV remote sensing imagery characterized by extreme scale diversity and high inter-class variability. Standard architectures often suffer from a rigid trade-off between local detail extraction and global context perception. This lack of adaptivity prevents the network from dynamically recalibrating its feature extraction strategies, leading to suboptimal cross-scale information fusion and a loss of critical semantic cues. Furthermore, the inherent computational redundancy in dense, fixed-kernel convolutions often compromises the balance between representation capacity and inference efficiency.

**Figure 3:**
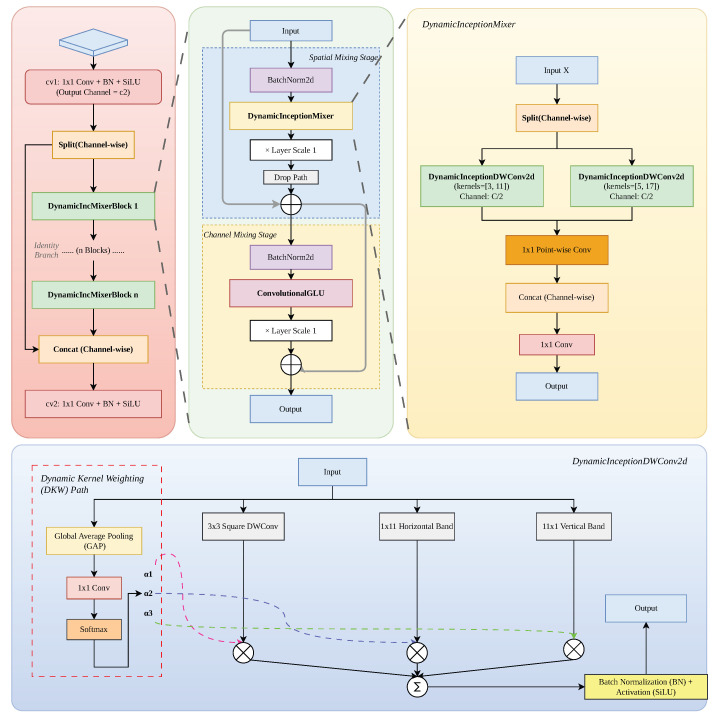
**Structural architecture of the proposed Dynamic Inception Mixer Block (C3k2-DIMB).** (**Top-left**) The macro-topology follows the Cross Stage Partial (CSP) strategy to optimize gradient flow and reduce memory footprint by splitting channels (C→C/2). (**Top-center**) The Spatial-to-Channel Mixing hierarchy, where Layer Scale (initialized at $10^{-2}$) and DropPath are integrated to ensure numerical stability in deep architectures. (**Top-right**) The structure of DynamicInceptionMixer. (**Bottom**) The DynamicInceptionDWConv2d operator, featuring the dynamic kernel weighting (DKW) path. It adaptively modulates a composite receptive field by fusing square (3×3) and band (1×11,11×1) kernels using softmax-generated attention coefficients ($\alpha_{1},\alpha_{2},\alpha_{3}$), specifically designed to resolve the geometric anisotropy of targets like vehicles and bridges (AR > 3) in UAV imagery.

To address these challenges, we propose the C3k2-DIMB module, which leverages a Dynamic Inception Mixer (DIM) to introduce a flexible, data-dependent convolutional paradigm. The structure of C3k2-DIMB is shown in Figure 3. By integrating a dynamic kernel weighting (DKW) mechanism, the DIM adaptively calibrates the contributions of heterogeneous depthwise convolutions—including square, horizontal, and vertical band kernels—based on input feature characteristics. This design enables the network to dynamically modulate its receptive field and capture multi-scale structural cues while maintaining a lightweight computational profile. Furthermore, the incorporation of Layer Scale and DropPath strategies within a residual learning framework ensures stable gradient flow and robust cross-layer information fusion. Consequently, C3k2-DIMB effectively bridges the gap between local fine-grained textures and global contextual semantics, significantly bolstering the network’s discriminative power for targets under varying scales.

Unlike conventional static convolutions, the DIM incorporates a data-dependent weight allocation mechanism. For an input feature map $X\in R^{C\times H\times W}$, the dynamic kernel weighting (DKW) first generates a global context descriptor $z\in R^{C}$ via global average pooling. A lightweight controller then computes a set of dynamic coefficients α through a softmax function:

(6) $$\alpha_{g}=\frac{\exp(W_{g}z)}{\sum_{j=1}^{G}\exp\left(W_{j}z\right)},\;g\in \left\{1,2,3\right\}$$

where $W_{g}$ denotes the learnable parameters of the control branch and G=3 represents the three heterogeneous kernels. The calibrated output $Y_{cal}$ is obtained by a weighted summation of the parallel branches:

(7) $$Y_{cal}=\mathrm{Act}\left(\mathrm{BN}\left(\sum_{g=1}^{G}\alpha_{g}\;\cdot \;{\mathrm{DWConv}}_{g}\left(X\right)\right)\right)$$

This mechanism allows the network to adaptively emphasize specific geometric patterns based on the input content.

To capture the diverse morphological characteristics of small targets with minimal overhead, we employ the idea of asymmetric convolution. In the DynamicInceptionDWConv2dlayer, this module decomposes a large-scale receptive field into a square kernel $K_{sq}\in R^{k\times k}$ and two orthogonal band kernels $K_{hor}\in R^{1\times n}$ and $K_{ver}\in R^{n\times 1}$. The effective receptive field ${\mathrm{RF}}_{eff}$ of this assembly is approximated as follows:

(8) $${\mathrm{RF}}_{eff}\approx \mathrm{RF}\left(K_{sq}\right)\cup \mathrm{RF}\left(K_{hor}\right)\cup \mathrm{RF}\left(K_{ver}\right)$$

By setting n=3k+2, the module simulates an expansive receptive field while significantly reducing the parameter count *P* compared to a standard n×n kernel:

(9) $$P_{asym}=C\times \left(k^{2}+2n\right)\ll C\times n^{2}$$

This design is particularly effective for UAV imagery, as it enhances boundary sensitivity for elongated targets at an extremely low computational cost. In this study, elongated targets are defined as objects exhibiting high geometric anisotropy, characterized by a significant disparity between their principal spatial dimensions. Specifically, we define a target as elongated when its aspect ratio (AR)—the ratio of the long axis to the short axis of its bounding box—exceeds a threshold (e.g., AR>3). Such targets, common in UAV imagery as ships, bridges, or vehicles viewed from oblique angles, pose a challenge for standard isotropic kernels (3×3) due to the morphological mismatch between the square receptive field and the target’s linear spatial extent.

To optimize the balance between representational diversity and inference efficiency, the C3k2-DIMB adopts a channel-splitting strategy. The input feature map *X* is partitioned into two distinct groups along the channel dimension:

(10) $$X=\left[X_{group1},X_{group2}\right],\;X_{group\_i}\in R^{\frac{C}{2}\times H\times W}$$

Each group is processed by independent DIM units with different kernel configurations. The transformed features are then aggregated using a 1×1 pointwise convolution to facilitate cross-channel interaction:

(11) $$X_{mixed}={\mathrm{Conv}}_{1\times 1}\left(\mathrm{Concat}\left[{\mathrm{DIM}}_{1}\left(X_{group1}\right),{\mathrm{DIM}}_{2}\left(X_{group2}\right)\right]\right)$$

This grouped mixing approach fosters high-dimensional feature diversity without incurring excessive memory traffic.

To ensure stable gradient propagation in deep architectures, this module introduces the Layer Scale mechanism [43], which is employed to modulate the residual branch. The output $X_{out}$ of the DynamicIncMixerBlock is calculated by modulating the residual branch with a learnable diagonal matrix $\Gamma =\mathrm{diag}(\gamma_{1},\gamma_{2},\ldots ,\gamma_{C})$:

(12) $$X_{out}=X+\mathrm{DropPath}\left(\Gamma \;\cdot \;\mathrm{Mixer}(\mathrm{Norm}(X))\right)$$

where $\gamma_{i}$ is initialized to $10^{-2}$. Furthermore, a Convolutional Gated Linear Unit (C-GLU) is employed in the feed-forward branch to filter irrelevant background noise, expressed as follows:

(13) $$Y_{G}=\left(X_{in}*W_{1}\right)⊙\sigma \left(X_{in}*W_{2}\right)$$

where * denotes convolution, ⊙ represents the element-wise product, and σ is the activation function. This non-linear gating mechanism significantly bolsters the model’s robustness in complex UAV scenarios.

### 3.4. Shuffled Upsampling for Resolution Enhancement Block

Traditional upsampling operators in deep neural networks, such as bilinear interpolation and transposed convolution, face significant limitations in UAV-based small object detection. Content-agnostic interpolation often leads to the dilution of spatial details and semantic blurring, making it difficult to reconstruct the impoverished geometric features of micro-scale targets. Conversely, while learnable transposed convolutions can capture more complex patterns, they frequently introduce checkerboard artifacts and impose a heavy computational burden, which is incompatible with the real-time requirements of edge-deployed UAV platforms. Furthermore, these conventional methods lack an integrated mechanism to effectively refine upsampled features or facilitate cross-channel information interaction, resulting in a loss of discriminative power for dense, small-scale objects amidst complex remote sensing backgrounds.

**Figure 4:**
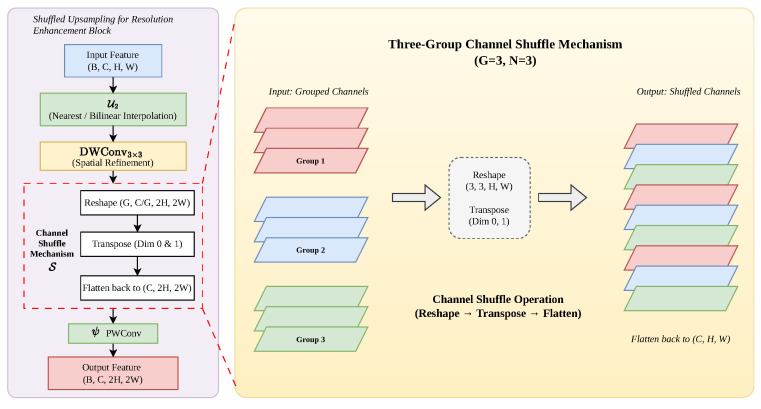
**Structural architecture of SURE module.** (**Left**) The overall pipeline comprises a nearest-neighbor upsampling operator $U_{2}$, a depthwise convolution ($DWConv_{3\times 3}$) for spatial refinement, the proposed channel shuffle mechanism S, and a final pointwise convolution ψ. (**Right**) An isometric visualization of the three-group channel shuffle operation (G=3,N=3). The process demonstrates how input features are restructured through reshape and transpose operations to facilitate cross-channel semantic interaction and break information isolation.

To overcome these challenges, this study proposes the SURE, whose structure is shown in Figure 4. The SURE addresses the limitations of standard operators by integrating a resolution-restoring upsampling mechanism with a lightweight Depthwise Separable Convolution framework. Specifically, the depthwise convolution (3×3) following the upsampling layer mitigates the blurring effect of standard interpolation, effectively refining the geometric features of tiny targets. Simultaneously, the inclusion of a channel shuffle operation overcomes the inherent information isolation of depthwise convolutions, facilitating robust cross-channel feature fusion without imposing additional parameter overhead. This synergy ensures that RFE-YOLO maintains high discriminative power for dense small objects while meeting the real-time requirements of UAV platforms.

Given an input feature map $X\in R^{C\times H\times W}$, where *C*, *H*, and *W* denote the number of channels, height, and width, respectively, the forward pass of the SURE is formulated as follows:

First, the spatial resolution is expanded via an upsampling operator U with a scale factor of 2, followed by a depthwise convolution (DWConv) for spatial feature refinement:

(14) $$X_{dw}={\mathrm{DWConv}}_{3\times 3}\left(U_{2}\left(X\right)\right)$$

where $X_{dw}\in R^{C\times 2H\times 2W}$. The operation for each channel *c* can be expressed as follows:

(15) $$X_{dw}^{\left(c\right)}=X_{up}^{\left(c\right)}*K^{\left(c\right)},\;c=1,2,\ldots ,C$$

where * denotes the convolution operation and $K^{\left(c\right)}$ represents the *c*-th depthwise kernel.

Standard interpolation (e.g., bilinear) is content-agnostic, often leading to semantic blurring. This module couples a nearest-neighbor upsampling with a depthwise convolution (DW Conv). By applying a learnable 3×3 filter to each channel independently, the DW Conv acts as a local feature refiner that suppresses the blurring effects of interpolation. This allows the network to adaptively “sharpen” the geometric boundaries of small objects, effectively restoring the spatial fidelity lost during the resolution enhancement.

A common drawback of lightweight designs is “information isolation,” where depthwise convolutions fail to exchange information across channels. To break the “information silos” caused by the depthwise operation, a channel shuffle mechanism [44] S is applied by reshaping and transposing the feature maps:

(16) $$X_{shuf}=S\left(X_{dw}\right)$$

Let the feature map after the upsampling and depthwise convolution layers be denoted as $X\in R^{C\times H\times W}$. This module partitions the *C* channels into *g* groups, with each group containing n=C/g channels. At each spatial position (h,w), the channel vector is reshaped into a matrix $M\in R^{g\times n}$, where the element $M_{i,j}$ corresponds to the (i·n+j)-th channel.

The shuffle operation is mathematically equivalent to a matrix transposition followed by a flattening operation. The index mapping function F that transforms the input channel index *k* to the output index $k^{\prime }$ is defined as follows:

(17) $$k^{\prime }=\left(k\;\mathrm{mod}\;g\right)\;\cdot \;\frac{C}{g}+\left\lfloor \frac{k}{g}\right\rfloor$$

The parameter *g* in Equation (18) denotes the number of groups for the shuffled upsampling process. The selection of *g* follows three primary scientific criteria: (1) Divisibility: *g* must be a common divisor of the input channels *C* to ensure symmetric feature distribution. (2) Information Capacity: While a larger *g* significantly reduces FLOPs, it may lead to “information isolation” between groups. To mitigate this, *g* is typically set to a small power of 3 (e.g., g∈{2,4}) in our RFE-YOLO to maintain high-fidelity cross-channel interaction for micro-targets. (3) Hardware Efficiency: Most edge-computing platforms (e.g., NVIDIA Jetson) are optimized for memory alignment in blocks of 2 or 4, making these values optimal for real-time inference speed.

This permutation ensures that the subsequent 1×1 pointwise convolution ψ aggregates features derived from disparate groups:

(18) $$Y_{c,h,w}=\sum_{k=0}^{C-1}W_{c,k}\;\cdot \;X_{F(k),h,w}$$

where W represents the learnable weights. This mechanism facilitates robust global–local feature fusion, which is vital for accurately localizing micro-scale objects in complex UAV remote sensing scenes.

Finally, a pointwise convolution (PWConv) is employed to aggregate the shuffled features across channels:

(19) $$Y=W_{pw}⊛X_{shuf}+b$$

where ⊛ denotes the 1×1 convolution and $W_{pw}\in R^{C\times C\times 1\times 1}$ is the learnable weight matrix. The complete mapping function of the SURE can be summarized as follows:

(20) $$Y=\psi \left(S\left(\mathrm{DWConv}\left(\mathrm{Up}\left(X\right)\right)\right)\right)$$

where ψ denotes the pointwise convolution operation.

Unlike transposed convolutions that frequently suffer from uneven overlaps (checkerboard artifacts), the SURE utilizes a smooth Upsample followed by a stride-1 convolution. This sequence ensures a more homogeneous distribution of pixel gradients, providing a more stable and cleaner feature representation for the subsequent detection heads.

### 3.5. Multi-Feature Fusion Module

The conventional *Concat* operation in YOLO11 performs a rigid, channel-wise stacking that treats heterogeneous feature maps with equal priority, which is suboptimal for the high-precision demands of UAV-based small object detection. In the complex backgrounds, this static fusion mechanism leads to the dilution of weak signals from micro-scale targets amidst massive redundant environmental information. Furthermore, the lack of an adaptive selection process fails to bridge the semantic gap between multi-scale layers, resulting in indiscriminate noise propagation and semantic misalignment that ultimately compromise localization accuracy and increase computational redundancy for edge-deployed UAV platforms.

**Figure 5:**
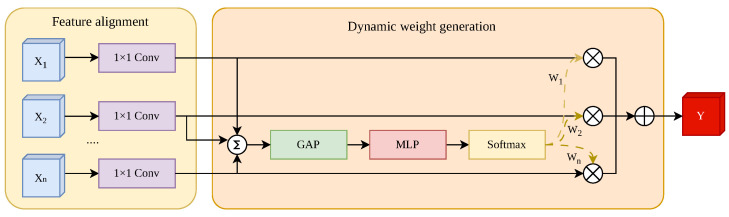
Structural architecture of the Multi-feature Fusion Module (MFM). The module consists of two main stages: (**Left**) Feature alignment, where input features $X_{1},\ldots ,X_{n}$ are projected into a unified channel space via 1×1 convolutions. (**Right**) Dynamic weight generation, which employs a softmax-based competition strategy. The aligned features are aggregated and passed through global average pooling (GAP) and an MLP to produce dynamic weights $w_{1},\ldots ,w_{n}$. These weights adaptively modulate the contribution of each branch for the final feature fusion Y.

To address the limitations of the static Concatoperation in YOLO11, this paper proposes the Multi-feature Fusion Module (MFM), and its structure is shown in Figure 5. Unlike the rigid stacking of features, MFM introduces a dynamic competition mechanism that adaptively weights multi-scale features based on their contribution to the detection task. The execution flow and mathematical formulation of the MFM are described below according to the sequential logic of the implementation:

The module first receives a set of *n* input feature maps $\left\{X_{1},X_{2},\ldots ,X_{n}\right\}$ from different levels of the network. To resolve the dimensionality mismatch (semantic gap), a set of 1×1 convolutions, denoted as $\phi_{i}$, is applied to align the channel dimensions to a target dimension *D*:

(21) $${\hat{X}}_{i}=\phi_{i}\left(X_{i}\right),\;i\in \left\{1,2,\ldots ,n\right\}$$

where ${\hat{X}}_{i}\in R^{D\times H\times W}$ represents the aligned feature map. In the case where the input dimension matches *D*, $\phi_{i}$ acts as an identity mapping.

Instead of treating features in isolation, MFM aggregates the information from all branches to form a holistic representation. The aligned features are integrated via an element-wise summation, followed by a global average pooling (P) to extract the global spatial context vector $s\in R^{D\times 1\times 1}$:

(22) $$s=P\left(\sum_{i=1}^{n}{\hat{X}}_{i}\right)$$

By compressing the spatial descriptors, the module gains a global receptive field, which is essential for identifying micro-scale targets amidst complex terrestrial clutter in UAV imagery.

The aggregated context s is processed by a bottleneck-structured Multi-Layer Perceptron (MLP) with a reduction ratio *r*. This generates branch-specific attention weights. To create a competitive environment among the *n* input branches, a Softmax function is applied along the branch dimension:

(23) $$A=\sigma \left(W_{2}\;\cdot \;\delta \left(W_{1}\;\cdot \;s\right)\right)$$

where $W_{1}\in R^{(D/r)\times D}$ and $W_{2}\in R^{\left(n\;\cdot \;D)\times (D/r\right)}$ are learnable weights, δ denotes the ReLU activation, and σ represents the Softmax operator. The resulting attention tensor A provides a specific weight $\alpha_{i}$ for the *i*-th input branch.

The final output Y is generated through an adaptive weighted summation of the input features. The Softmax normalization ensures that $\sum_{i=1}^{n}\alpha_{i}=1$, effectively prioritizing high-resolution spatial details for tiny objects while suppressing redundant noise from cluttered backgrounds:

(24) $$Y=\sum_{i=1}^{n}A_{i}⊙{\hat{X}}_{i}$$

where ⊙ denotes element-wise multiplication. This closed-loop design ensures that RFE-YOLO can dynamically bridge the semantic gap between multi-scale layers.

## 4. Experiments and Analysis

In this chapter, we conduct a series of comprehensive experiments to evaluate the performance of the proposed RFE-YOLO. The primary objective is to validate whether the integration of CSRE, C3k2-DIMB, SURE, and MFM can effectively address the challenges of micro-scale object detection in complex UAV remote sensing scenarios. The design of RFE-YOLO is based on the YOLO11n architecture, but its core components—the CSRE, C3k2-DIMB, and MFM modules are structurally modular. This implies that the proposed enhancements are not restricted to YOLO11 and can be seamlessly integrated into earlier versions (e.g., YOLOv8 or YOLOv10). The choice of YOLO11n as the baseline is motivated by its status as the advanced iteration in the YOLO lineage, providing a high-performance benchmark for evaluating structural intelligence. Furthermore, RFE-YOLO is specifically engineered for the nano-scale computational budget, achieving superior precision with fewer parameters (1.91 M) than the standard YOLO11n (2.58 M), thereby demonstrating its extreme efficiency for edge-constrained UAV deployment. The following sections detail the experimental setup, comparative analysis with state-of-the-art (SOTA) models, and an extensive ablation study to verify the contribution of each individual module.

### 4.1. Datasets and Experimental Parameters

The VisDrone2019 dataset, curated by the AISKYEYE team at Tianjin University, serves as an authoritative benchmark for evaluating object detection performance in UAV-based remote sensing scenarios. It comprises 10,209 high-resolution images captured under diverse weather and lighting conditions, which are officially partitioned into training (6471 images), validation (548 images), and testing (1580 images) sets. This dataset encompasses ten distinct categories, such as pedestrians, cars, and vans, and is characterized by exceptionally high target density and a large proportion of micro-scale objects. Specifically, over 60% of the targets are small objects with a pixel area less than 32 × 32, providing a rigorous test for the lossless information preservation capabilities of our proposed CSRE module.

While the inherent challenges of the VisDrone dataset—such as extreme scale variations, high occlusion, and low-resolution micro-targets—were highlighted, it remains a popular benchmark in the UAV detection community, and our decision to utilize it was strategically motivated. Beyond facilitating a rigorous and fair performance comparison against existing state-of-the-art models within a standardized framework, the very “defects” of the dataset provide a robust stress-testing environment to validate the efficacy of RFE-YOLO. By achieving superior results under these demanding conditions, we demonstrate that the proposed CSRE and DIM modules specifically mitigate the information loss and geometric distortion characteristic of such difficult sensing scenarios, effectively bridging the gap between our initial critical analysis and the subsequent experimental validation.

In this paper, the original dataset preset ratio is used to divide the training, validation, and test sets. Our experiments are conducted on an Intel Xeon W-2245 CPU and an RTX3090 24G GPU, with Ubuntu 20.04, PyTorch 1.11, and CUDA 11.3. For the parameter setting in the training stage, this study removes the left–right flip augmentation based on the official training of YOLOv5 from scratch, the batch size is 8, the training is 300 epochs, and the image size is 640. Table 1 details the main parameter settings used during training.

### 4.2. Ablation Study and Performance Analysis

To verify the “Preservation–Adaptation and Fusion” hypothesis, an incremental ablation study was conducted on the VisDrone2019 dataset. The results provide a quantitative validation of the theoretical motivations behind each proposed component, including Precision (P), Recall (R), $mAP_{50}$, $mAP_{50:95}$, Parameters (M), and FLOPs (G), as summarized in Table 2.

This study utilizes YOLO11 as the primary baseline, which achieves an $mAP_{50}$ of 33.40%. Given the prevalence of micro-scale targets in UAV imagery, this study also considers YOLO11+p2-p5 (integrating a P2 high-resolution feature layer), which increases the $mAP_{50}$ to 36.40% but at the cost of higher computational complexity (9.8 G FLOPs). The final model, RFE-YOLO, significantly outperforms this enhanced baseline, reaching $42.70\%\;mAP_{50}$ and $22.93\%\;mAP_{50\text{-}95}$, representing a substantial improvement of 6.3% and 3.11%, respectively.

#### 4.2.1. Individual Module Significance: Addressing Sensing Bottlenecks

The initial phase of the ablation study highlights the scientific necessity of specialized operators in micro-target detection:

Information Preservation via CSRE (Scheme 1): The introduction of the CSRE module yields the most significant single-module gain ($+2.81\%\;mAP_{50}$ over the baseline). This result scientifically validates the premise that irreversible information attrition caused by periodic pixel discarding in standard strided convolutions is a primary bottleneck. By migrating spatial details into the channel dimension via SPD-Conv and purifying signals through energy-based weighting, CSRE maintains the structural integrity of targets with limited pixel support.

Geometric Adaptation via C3k2-DIMB (Scheme 2): The improvement to $37.53\%\;mAP_{50}$ reinforces the need for anisotropic modeling. Unlike the morphological rigidity of static kernels, the dynamic inception kernels in DIMB allow the network to calibrate its receptive field shape to the specific aspect ratios of elongated targets, effectively handling the geometric distortions common in variable-altitude UAV imagery.

High-Fidelity Restoration via SURE and MFM (Schemes 3 and 4): The gains from SURE (37.80%) and MFM (37.20%) demonstrate that detection performance is highly sensitive to the quality of the “Extraction–Restoration” loop. SURE breaks the information silos inherent in depthwise operations through the channel shuffle mechanism S, while MFM acts as a dynamic filter, utilizing a softmax-based competition strategy to prioritize discriminative signals over terrestrial background clutter.

#### 4.2.2. Synergistic Effects: The Preservation–Restoration Loop

The dual and triple-module configurations reveal critical inter-modular dependencies that define the model’s robustness:

The “Extraction–Restoration” Synergy (Scheme 6): The combination of CSRE and SURE achieves $40.80\%\;mAP_{50}$, the highest among dual-module schemes. This synergy is scientifically significant as it completes a high-fidelity spatial pipeline: CSRE prevents information loss during downsampling, while SURE ensures that this preserved information is reconstructed without semantic blurring during resolution recovery.

Semantic Purification and Competition (Scheme 12): The contrast in Scheme 12 (high Precision but lower Recall) suggests that while a high-fidelity spatial pipeline (CSRE+SURE) ensures accurate localization, the lack of adaptive receptive fields (C3k2-DIMB) limits the model’s ability to “fit” diverse object morphologies. The addition of MFM ensures that multi-scale features are semantically purified before the final detection stage.

The Semantic Bridge (Scheme 14): The jump to $42.20\%\;mAP_{50}$ demonstrates that MFM acts as a vital bridge between high-resolution spatial details and abstract semantics. By dynamically weighting multi-scale features, MFM ensures that the fusion process is semantically concentrated, leading to a significant gain over the +p2-p5 baseline.

#### 4.2.3. Final Integration and Research Conclusion

The full integration in RFE-YOLO culminates in an optimal $mAP_{50}$ of 42.70% and a Recall (*R*) of 38.78%. The steady rise in Recall across all schemes (33.34%→38.78%) indicates a significant reduction in false negatives, particularly for dense, micro-scale targets.

The marginal gain from Scheme 14 to the full model suggests that C3k2-DIMB provides the final geometric refinement. Ultimately, the results prove that structural intelligence—characterized by lossless migration, dynamic adaptation, and competitive fusion—can effectively replace brute-force parameter stacking, achieving superior representation capacity with a remarkably lightweight profile of 1.91 M parameters.

### 4.3. Comparative Experiment

To validate the superiority of the proposed RFE-YOLO, we conducted a horizontal comparison with several state-of-the-art (SOTA) object detection models on the VisDrone2019 dataset. The results, summarized in Table 3, evaluate each model based on detection accuracy ($mAP_{50}$, $mAP_{50\text{-}95}$), recall (*R*), and computational efficiency (Parameters and FLOPs).

The experimental results demonstrate that RFE-YOLO achieves the highest detection accuracy among all compared models. Specifically, our model reaches an $mAP_{50}$ of 42.7%, outperforming the baseline YOLO11n (33.4%) and the recent YOLO13n (32.96%) by a substantial margin of +9.3% and +9.74%, respectively.

The substantial accuracy leap over the baseline YOLO11n ($+9.3\%\;mAP_{50}$) is scientifically significant as it validates the hypothesis that pixel-level information preservation is more critical than raw network depth for micro-scale target detection. While standard models suffer from irreversible feature attrition during downsampling, the RFE-YOLO’s CSRE module ensures that spatial details are migrated rather than discarded. This suggests that the proposed “Preservation–Adaptation” strategy effectively mitigates the intrinsic resolution bottlenecks of standard convolutional architectures in remote sensing scenarios.

Furthermore, RFE-YOLO demonstrates significant superiority over specialized UAV-centric models such as TPH-yolov5-s (37.4% $mAP_{50}$) and Drone-YOLO (38.1% $mAP_{50}$). Notably, our model also yields the highest Recall rate of 38.78%, which is critical for UAV applications to minimize the omission of micro-scale objects in dense urban environments. In the context of UAV applications, this improvement is indicative of superior signal-to-noise discrimination. The synergy between the SURE and MFM modules allows the network to effectively bridge the semantic–spatial gap, ensuring that minute targets are distinguished from complex terrestrial clutter rather than being suppressed as background noise. The high $mAP_{50\text{-}95}$ score of 22.93% further validates the precise localization capabilities of RFE-YOLO, exceeding the highly complex SCA-DEIM-S (23.4% $mAP_{50\text{-}95}$) despite having significantly lower computational overhead.

A key highlight of RFE-YOLO is its remarkable parameter efficiency. As shown in Table 3, our model contains only 1.91 M parameters (normalized from 1,908,722), making it lighter than the baseline YOLO11n (2.58 M) and nearly 5.4 times smaller than SCA-DEIM-S (10.42 M).

Regarding computational complexity, while RFE-YOLO incurs 13.5G FLOPs—higher than the standard YOLO11n (6.3 G)—it remains considerably more efficient than models with similar accuracy levels, such as SRM-YOLO (15.9 G) and LW-yolov8 (22 G). This “parameter–performance paradox” is attributed to our strategic use of depthwise convolutions in the C3k2-DIMB and SURE modules, which replace redundant parameter-heavy layers with efficient, cross-channel information-sharing mechanisms.

To ensure a comprehensive and persuasive evaluation, the state-of-the-art (SOTA) models selected for comparison in Table 3 cover three representative paradigms in the UAV detection domain: (1) Standard Baselines, including the latest YOLO11n and YOLO13n, to benchmark the evolution of generic architectures; (2) UAV-Specific Models, such as TPH-yolov5-s and Drone-YOLO, which incorporate specialized heads or attention for aerial views; and (3) Efficiency-Oriented Models, such as DL-DEIM and LW-YOLOv8, which prioritize lightweight deployment. By achieving the highest $mAP_{50}$ (42.7%) with the lowest parameter count (1.91 M), RFE-YOLO demonstrates superior structural intelligence across all three categories. Regarding the missing metrics (indicated by “–”) for certain SOTA models, these were omitted to maintain scientific integrity, as the original authors did not report these specific values (e.g., P,R, or FLOPs), and local re-implementations may fail to reach their reported performance due to differing training hyperparameters.

In conclusion, the proposed RFE-YOLO achieves an optimal balance between accuracy and efficiency. By outperforming recent high-performance models like SRM-YOLO (+3.3% $mAP_{50}$) while reducing the parameter count by approximately 40%, our model proves to be a robust and highly deployable solution for real-time edge-constrained UAV platforms.

### 4.4. Experiment on Other Dataset

To demonstrate that the performance gains of RFE-YOLO are not confined to a specific experimental environment, we extended our evaluation to two structurally diverse datasets: NWPU VHR-10 [40] and UAVDT [39]. These results provide critical evidence of the model’s cross-domain robustness and its ability to resolve universal sensing bottlenecks.

Table 4 summarizes the performance on the NWPU VHR-10 dataset. RFE-YOLO achieves an overall $mAP_{50}$ of 89.6% and $mAP_{50\text{-}95}$ of 56.5%, outperforming the baseline by +3.9% and +4.6%, respectively.

A striking improvement is observed in the basketball court category ($+13.9\%\;mAP_{50}$) and vehicle ($+10.2\%\;mAP_{50}$). Unlike standard isotropic kernels that struggle with the high aspect ratios of courts and the minute scale of vehicles, the C3k2-DIMB module adaptively modulates its receptive field. This validates that the dynamic inception mechanism is highly effective at capturing diverse geometric signatures across different imaging altitudes.

Significant gains in $mAP_{50\text{-}95}$ for categories like harbor (+10.8%) and airplane (+8.6%) suggest that the SURE and MFM modules successfully suppress boundary blurring. By recovering spatial resolution with high fidelity, RFE-YOLO ensures precise edge delineation even when targets are situated against complex terrestrial backgrounds, such as runways or docks.

In terms of robustness across scales, the consistent improvement across both large-scale structures (e.g., ground track field) and small-scale objects (e.g., storage tank) proves that the CSRE module’s lossless information migration provides a more stable feature foundation than traditional strided downsampling.

To further test the model under varying lighting and traffic conditions, evaluations were performed on the UAVDT dataset (Table 5). RFE-YOLO maintained a consistent lead over the baseline with a +3.4% increase in $mAP_{50}$ and a +2.4% increase in $mAP_{50\text{-}95}$.

UAVDT is characterized by intense traffic density and varying illumination. The steady performance gain indicates that the modules in this paper effectively filter out environmental noise, ensuring that the model remains focused on target-specific discriminative features. The synchronized performance growth across VisDrone (Urban), NWPU (Satellite), and UAVDT (Traffic) datasets demonstrates that RFE-YOLO addresses the intrinsic physical limitations of convolutional sensing rather than merely overfitting to dataset-specific biases. This confirms the proposed architecture as a robust, universal solution for high-precision detection on edge-deployed aerial platforms.

### 4.5. Visualization Experiments

**Figure 6:**
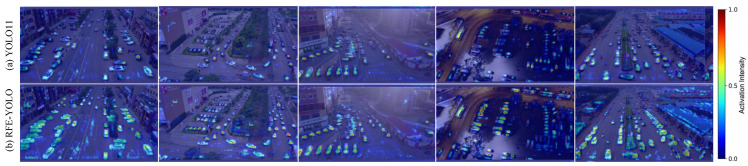
Visualization comparison using heatmap activation mapping on the VisDrone2019 dataset. (**a**) Baseline YOLO11 results showing diffused activations with significant background overlap. (**b**) Proposed RFE-YOLO results showing concentrated and discretized activations, demonstrating high-fidelity localization and individual target delineation. The numerical color bar represents the normalized activation intensity, where values range from 0.0 to 1.0 to indicate the degree of feature concentration, with 1.0 signifying the peak response on target-specific regions.

To verify the scientific rationale behind the performance gains, feature heatmap activation mapping is utilized to intuitively visualize the models’ localization and discrimination capabilities. Figure 6 compares the baseline YOLO11 (left column) and our RFE-YOLO (right column) across five representative scenarios, including a complex street corner, a dense parking lot, and diverse urban streets.

As demonstrated in the YOLO11 results (Figure 6a), feature activations are consistently diffused and exhibit activation overlap between adjacent targets. In dense scenes (rows 1, 4), YOLO11 fails to produce heatpoints on individual targets. Instead, activations are either weak, concentrated only on target edges, or create a continuous, non-discriminative block across multiple targets. This pattern confirms that traditional content-agnostic downsampling and upsampling in the baseline model lead to significant information isolation and semantic blurring, restricting the model’s ability to precisely delineate micro-scale object boundaries in cluttered remote sensing scenes.

In contrast, RFE-YOLO (Figure 6b) generates concentrated, discretized activation patterns. In the same scenarios, our model produces clearly defined, high-intensity heatpoints on each independent target, covering a larger, more relevant portion of the target’s body. This significant improvement in localization precision is most pronounced in complex viewpoints (rows 2 and 4), where targets are densely packed with varying orientations. RFE-YOLO maintains distinct activations on separate objects, preventing region-based diffusion.

The optimized feature activations observed in the proposed architecture are scientifically attributed to the synergistic integration of the Cross-Scale Receptive Field Enhancement (CSRE), Dynamic Inception Mixer Block (DIMB), and Multi-feature Fusion Module (MFM). Specifically, the CSRE module facilitates pixel-level detail preservation through spatial statistics-based energy function recalibration and space-to-depth convolution, which effectively mitigates the information loss inherent in standard downsampling and results in precise, localized activations on micro-scale objects rather than diffused feature blobs. This spatial fidelity is further augmented by the DIMB, which enables geometric adaptation through dynamic inception kernels that adaptively modulate the receptive field shape to accurately model anisotropic distortions and maintain concentrated activations across complex viewpoints and varying target orientations. Finally, the MFM ensures robust cross-scale integration by utilizing a softmax-based competition strategy to prioritize discriminative features, thereby dynamically fusing high-resolution spatial details with deep contextual cues to produce a purified and high-intensity signal across all detection scales.

The discretization and precision of RFE-YOLO’s activations directly correlate with and explain the significant numerical gains observed in the ablation study (Table 2). Specifically, by producing discretized activations, especially on small objects like pedestrians in crowded areas (row 4), RFE-YOLO facilitates robust detection and accurate counting of dense targets. This localized discriminative power directly supports the observed increase in Recall (33.34% to 38.78%) and specific small targets (Pedestrian AP from 35.9% to 44.4%), thus resolving the common “information isolation” and “semantic blurring” issues of lightweight designs in UAV applications.

**Figure 7:**
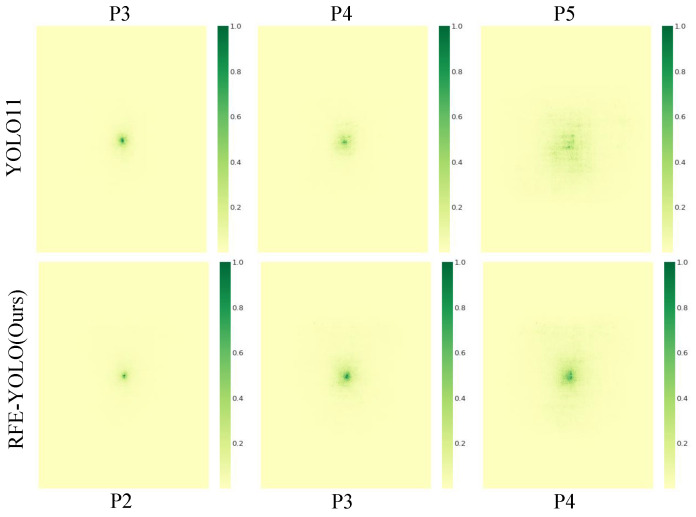
Effective receptive field (ERF) visualization comparison. The first row displays the relatively diffused and scattered ERF patterns of the baseline YOLO11 across the P3, P4, and P5 layers, indicating a potential struggle to delineate precise object boundaries. The second row illustrates the significantly more centralized and focused ERF patterns of the proposed RFE-YOLO across the P2, P3, and P4 layers.

To demonstrate the architectural superiority of RFE-YOLO in focusing on micro-scale targets, the effective receptive fields (ERFs) are visualized at various detection scales as shown in Figure 7, where the first row displays the ERF of the baseline YOLO11 across the P3, P4, and P5 layers with strides of 8, 16, and 32, respectively, and the second row illustrates the ERF of the proposed RFE-YOLO for the P2, P3, and P4 layers with strides of 4, 8, and 16. The integration of the high-resolution P2 layer enables RFE-YOLO to achieve a significantly smaller and more refined effective receptive field compared to the baseline, which proves highly advantageous for the detection of micro-scale objects such as pedestrians and distant vehicles by ensuring the network focuses precisely on the target’s spatial extent without being overwhelmed by background clutter. At equivalent scales, specifically within the P3 and P4 layers, the ERF of RFE-YOLO exhibits a noticeably higher concentration toward the center region compared to the diffused and scattered patterns observed in the YOLO11 baseline, indicating that the proposed CSRE and C3k2-DIMB modules effectively calibrate the receptive field to facilitate the extraction of more discriminative features. This centralized focus enhances the signal-to-noise ratio for small objects and directly correlates with the substantial improvements in $mAP_{50}$ and recall observed in the quantitative experimental results.

## 5. Conclusions and Future Work

### 5.1. Conclusions

This research proposed RFE-YOLO, a lightweight high-performance object detection framework specifically engineered for micro-scale target detection in complex UAV remote sensing scenarios. Our work addresses the critical challenges of information isolation and semantic blurring that often compromise the effectiveness of standard architectures in high-density, micro-scale environments. The following conclusions are drawn from our theoretical design and extensive experimental validation:

1. Architectural Innovation: We successfully established a “preservation–adaptation–reconstruction” pipeline. The CSRE module effectively mitigates pixel-level information loss during downsampling; the C3k2-DIMB adaptively modulates the receptive field using dynamic inception kernels to handle anisotropic distortions; the SURE module restores spatial fidelity via a channel shuffle mechanism; and the MFM bridges the semantic gap through a softmax-based competition strategy.
2. Superior Performance and Efficiency: Experimental results on the VisDrone2019 and UAVDT datasets demonstrate that RFE-YOLO achieves a state-of-the-art $mAP_{50}$ of 42.7%, surpassing the baseline YOLO11n by +9.3%. Notably, our model maintains an exceptionally lightweight profile with only 1.91 M parameters—lower than the standard YOLO11n—proving that high-precision UAV surveillance can be achieved through structural intelligence rather than brute-force parameter stacking.
3. Qualitative and Structural Validation: The unified analysis of effective receptive fields (ERFs) and feature activation mapping confirms that RFE-YOLO possesses a highly centralized and focused receptive field. This structural morphology directly translates into discrete, high-intensity activation patterns on micro-targets, effectively suppressing background noise and resolving boundary confusion issues in dense urban scenarios.

### 5.2. Future Work

Despite the significant advancements demonstrated by RFE-YOLO, several avenues for future research remain:

1. Edge Hardware Optimization: Future work will focus on optimizing the model for specific embedded hardware platforms (e.g., NVIDIA Jetson, Rockchip NPU) to further reduce inference latency and power consumption for onboard drone processing.
2. Multimodal Feature Fusion: Integrating additional data sources, such as infrared (IR) or LiDAR point clouds, could enhance the model’s robustness in extreme low-light or adverse weather conditions where visible-light features are degraded.
3. Temporal Consistency in Video Streams: Expanding the current frame-based detection to incorporate temporal information across video sequences could improve the tracking and localization stability of fast-moving micro-objects.

## Figures and Tables

**Table 1:** The main parameter settings.

Parameters	Setup
Epochs	300
Batch size	8
Workers	8
Imgsz	640
Optimizer (SGD)	0.7
Initial learning rate	$1\times 10^{-2}$
Final learning rate	$1\times 10^{-4}$
Momentum	0.937
Mosaic	1
Warmup epochs	10
IoU	0.7

**Table 2:** Ablation study on VisDrone2019 dataset. **P**, **R**, and **mAP** are in (%). **Param** is in Millions (M), and **FLOPs** are in Giga (G). ✓ indicates that the corresponding module is in use.

Method	Components				Performance Metrics (%)				Param	FLOPs
	CSRE	C3k2	SURE	MFM	P	R	${\mathrm{mAP}}_{50}$	${\mathrm{mAP}}_{50\text{-}95}$	(M)	(G)
YOLO11					43.27	33.34	33.40	19.39	2.58	6.3
YOLOY11+p2-p5					43.50	33.61	36.40	19.82	1.94	9.8
1	✓				47.90	31.60	39.21	20.96	2.07	14.6
2		✓			45.30	32.80	37.53	20.17	1.86	11.8
3			✓		44.80	33.10	37.80	20.30	2.12	13.2
4				✓	44.10	33.90	37.20	20.10	2.06	12.4
5	✓	✓			47.20	34.50	40.10	21.50	1.90	13.9
6	✓		✓		48.20	33.80	40.80	21.90	2.17	15.3
7	✓			✓	47.60	34.20	39.90	21.40	2.10	14.3
8			✓	✓	45.50	34.80	38.60	20.80	1.95	12.5
9		✓		✓	45.80	34.40	38.20	20.50	1.88	11.7
10			✓	✓	46.10	35.00	38.90	21.00	2.15	13.1
11		✓	✓	✓	46.50	35.90	39.80	21.60	1.98	12.4
12	✓		✓	✓	48.60	36.50	41.70	22.40	2.19	15.0
13	✓	✓		✓	47.80	37.10	41.20	22.10	1.92	13.6
14	✓	✓	✓		48.30	37.90	42.20	22.60	1.99	14.6
**RFE-YOLO**	✓	✓	✓	✓	**48.13**	**38.78**	**42.70**	**22.93**	**1.91**	**13.5**

**Table 3:** Comparison of the proposed RFE-YOLO with state-of-the-art models on the VisDrone2019 dataset. The best results in each column are highlighted in **bold**. “–” indicates the data was not provided in the original paper.

Model	P (%)	R (%)	${\mathrm{mAP}}_{50}$ (%)	${\mathrm{mAP}}_{50\text{-}95}$ (%)	Params (M)	FLOPs (G)
YOLO11n	43.27	33.34	33.40	19.39	2.58	6.30
YOLO13n	43.10	32.82	32.96	19.24	2.45	**6.20**
TPH-yolov5-s [27]	–	–	37.40	21.70	–	–
Drone-YOLO [45]	–	–	38.10	22.70	3.50	–
DL-DEIM [46]	45.60	37.30	34.90	20.00	4.64	11.73
LW-YOLOv8 [47]	**51.20**	37.40	31.00	17.00	6.90	22.00
SCA-DEIM-S [48]	–	–	38.60	**23.40**	10.42	24.57
SRM-YOLO [49]	49.40	38.10	39.40	–	3.20	15.90
**RFE-YOLO (Ours)**	48.13	**38.78**	**42.70**	22.93	**1.91**	13.50

**Table 4:** Detection performance of YOLO11n and RFE-YOLO on the Northwestern Polytechnical University Very High Resolution-10 dataset.

Class	YOLO11n		Ours		Improvement	
	${\mathrm{AP}}_{50}$	${\mathrm{AP}}_{50\text{-}95}$	${\mathrm{AP}}_{50}$	${\mathrm{AP}}_{50\text{-}95}$	${\mathrm{AP}}_{50}$	${\mathrm{AP}}_{50\text{-}95}$
airplane	98.1	56.6	99.7	65.2	+1.6%	+8.6%
ship	85.7	59.3	89.3	59.3	+3.6%	+0.0%
storage tank	96.3	47.8	98.2	48.4	+1.9%	+0.6%
baseball diamond	93.7	72.0	93.9	72.3	+0.2%	+0.3%
tennis court	96.9	64.1	97.5	65.2	+0.6%	+1.1%
basketball court	36.8	25.6	50.7	33.5	+13.9%	+7.9%
ground track field	99.5	73.9	99.5	77.6	+0.0%	+3.7%
harbor	94.5	52.4	95.9	63.2	+1.4%	+10.8%
bridge	86.0	31.9	91.5	36.2	+5.5%	+4.3%
vehicle	69.2	35.7	79.4	43.6	+10.2%	+7.9%
All	85.7	51.9	89.6	56.5	+3.9%	+4.6%

**Table 5:** Detection performance of YOLO11n and RFE-YOLO on UAVDT dataset.

Class	YOLO11n		Ours		Improvement	
	${\mathrm{AP}}_{50}$	${\mathrm{AP}}_{50\text{-}95}$	${\mathrm{AP}}_{50}$	${\mathrm{AP}}_{50\text{-}95}$	${\mathrm{AP}}_{50}$	${\mathrm{AP}}_{50\text{-}95}$
All	32.4	20.0	35.8	22.4	+3.4%	+2.4%

## Data Availability

The data used in this analysis are publicly available, and access is provided in the text.
